# Supplementary material for: 'I fit the category of the box, it just doesn’t describe me well.' Exploring the perspectives of autistic women and gender-diverse individuals on self-report autism measures
Source: PLoS One. 2026 Jan 14;21(1):e0337600. doi: 10.1371/journal.pone.0337600 (PMC12803452; doi:10.1371/journal.pone.0337600)
Supplement: S1 Table — (PDF) [file pone.0337600.s001.pdf]

S1 Table. Interview guide.

No.	Question
1	Have you ever completed any of the following questionnaires before we sent them to you as a part of this study: AQ-10, RAADS-14, BAPQ?<br>a. *Follow-up question:* (If they said **YES** to question #1) What has been your experience with them?
2	Was there anything that you **particularly liked** about these questionnaires?
3	Was there anything that you **particularly disliked** about these questionnaires?
4	Were there any questions from these questionnaires that you found **particularly easy** to answer?
5	Were there any questions from these questionnaires that you found **particularly difficult** to answer?
6	Were there any questions you think were **missing** from these questionnaires?
7	Was there anything about the format of these questionnaires that made it particularly easy or difficult for you to complete them? For example, the response formats, length of the questionnaire, instructions.
8	(If they said **YES** to question #1) Did you feel that those questionnaires were helpful **(for diagnosed)** in the diagnostic process / **(for self-identifying)** in realising that you are autistic?
9	Do you think certain autistic traits present differently in your gender?<br>a. *Follow-up question*: What kind of questions should be asked to make sure they are relevant to your gender?
10	Are there any important factors to consider when assessing autism in relation to your gender? For instance, masking.<br>a. *Follow up question:* Do you think these questionnaires capture them?
11	Is there anything else you would like to tell us that we have not asked?
