# Supplementary material for: 'I fit the category of the box, it just doesn’t describe me well.' Exploring the perspectives of autistic women and gender-diverse individuals on self-report autism measures
Source: PLoS One. 2026 Jan 14;21(1):e0337600. doi: 10.1371/journal.pone.0337600 (PMC12803452; doi:10.1371/journal.pone.0337600)
Supplement: S2 Table — (PDF) [file pone.0337600.s002.pdf]

S2 Table. Demographic characteristics of participants (N=22).

Characteristic		Frequency (%)
**Gender identity**	Woman	[1] 17 (77.27)
	Agender	1 (4.55)
	Genderqueer	1 (4.55)
	Non-binary	2 (9.09)
	Other	1 (4.55)
**Ethnicity**	Asian/Asian British	6 (27.27)
	Black/African/Caribbean/Black British	6 (27.27)
	Mixed/multiple ethnic groups	3 (13.64)
	White/Caucasian	7 (31.82)
**Education**	Some secondary education	2 (9.09)
	Secondary school graduate	1 (4.55)
	Foundation degree	1 (4.55)
	Bachelor’s degree	11 (50.00)
	Master's or postgraduate degree	6 (27.27)
	Doctoral-level degree	1 (4.55)
**Autism**	Diagnosed	16 (72.73)
	Self-identifying	6 (27.27)
**Neurodivergence other than autism**	None	10 (45.50)
	Attention deficit hyperactivity disorder	8 (36.36)
	Dyslexia	3 (13.64)
	Dyspraxia	1 (4.55)
	Not sure	1 (4.55)

**Mental health conditions**	None	5 (22.73)
	Agoraphobia	1 (4.55)
	Complex post-traumatic stress disorder	1 (4.55)
	Depression	10 (45.45)
	Eating disorder not otherwise specified	1 (4.55)
	Generalised anxiety disorder	10 (45.45)
	Obsessive-compulsive disorder	2 (9.09)
	Panic disorder	1 (4.55)
	Post-traumatic stress disorder	5 (22.73)
	Selective mutism	1 (4.55)
	Social anxiety disorder	1 (4.55)
	Not sure	1 (4.55)
	Prefer not to say	2 (9.09)

Note. [1] All women participants identified as cis gender.
